# Association between maternal stress and premature milk cortisol, milk IgA, and infant health: a cohort study

**DOI:** 10.3389/fnut.2024.1270523

**Published:** 2024-03-06

**Authors:** Casey B. Rosen-Carole, Susan Greenman, Hongyue Wang, Sharvari Sonawane, Ravi Misra, Tom O'Connor, Kirsi Järvinen, Carl D'Angio, Bridget E. Young

**Affiliations:** ^1^Department of Pediatrics, University of Rochester School of Medicine and Dentistry, Rochester, NY, United States; ^2^Swedish First Hill Family Medicine, Seattle, WA, United States; ^3^Department of Psychiatry, University of Rochester School of Medicine and Dentistry, Rochester, NY, United States

**Keywords:** human milk, breastmilk, cortisol, stress, neurodevelopment, infant

## Abstract

**Background:**

Maternal stress is pervasive in the neonatal intensive care unit (NICU). Maternal stress is associated with changes in human milk (HM) immunomodulatory agents, which may impact neonatal health. We sought to determine the association between maternal stress, HM immunoglobulin A (IgA) and cortisol, and to assess how these milk components correlate with infant immune and neurodevelopmental outcomes. We then compared how these associations persist over time.

**Methods:**

The study design involved a cohort study of exclusively breastfeeding mothers and their singleton moderately preterm (28–34 weeks) infants admitted to the NICU. We collected maternal serum, maternal saliva, and first-morning whole milk samples, and administered maternal stress questionnaires at 1 and 5 weeks postpartum. We analyzed the samples for HM IgA (using a customized immunoassay in skim milk) and for HM and salivary cortisol (using a chemiluminescent immunoassay). Infant illness was assessed using the Score for Neonatal Acute Physiology II (SNAP II) and SNAP II with Perinatal Extension (SNAPPE II), and infant neurodevelopment were assessed using the Test of Infant Motor Performance. We analyzed changes in HM IgA and cortisol over time using paired *t*-tests. Furthermore, we performed correlation and regression analyses after adjusting for gestational age (GA), corrected GA, and infant days of life.

**Results:**

In our study, we enrolled 26 dyads, with a mean maternal age of 28.1 years, consisting of 69% white, 19% Black, and 8% Hispanic. Cortisol: Salivary and HM cortisol were closely associated in week 1 but not in week 5. Though mean salivary cortisol remained stable over time [2.41 ng/mL (SD 2.43) to 2.32 (SD 1.77), *p* = 0.17], mean HM cortisol increased [1.96 ng/mL (SD 1.93) to 5.93 ng/mL (SD 3.83), *p* < 0.001]. Stress measures were inversely associated with HM cortisol at week 1 but not at week 5. IgA: HM IgA decreased over time (mean = −0.14 mg/mL, SD 0.53, *p* < 0.0001). High maternal stress, as measured by the Parental Stressor Scale: neonatal intensive care unit (PSS:NICU), was positively associated with HM IgA at week 5 (r = 0.79, *P* ≤ 0.001). Higher IgA was associated with a lower (better) SNAP II score at week 1 (r = −0.74, *p* = 0.05). No associations were found between maternal stress, salivary cortisol, HM cortisol, or HM IgA and neurodevelopment at discharge (as assessed using the TIMP score). Furthermore, these relationships did not differ by infant sex.

**Conclusion:**

Maternal stress showed associations with HM cortisol and HM IgA. In turn, HM IgA was associated with lower measures of infant illness.

## Introduction

The provision of mother’s milk for sick neonates promotes infant feeding tolerance, growth, and neurodevelopment, while serving as a preventive measure against life-threatening diseases such as necrotizing enterocolitis (1). However, providing mother’s milk also adds to the family’s burden of stress during a tumultuous time (2, 3). Mothers in the neonatal intensive care unit (NICU) report high levels of stress, with more than 40% experiencing clinical depression by the time of NICU discharge (4). While the human milk (HM) quantity and the onset of lactogenesis 2 have been shown to be negatively impacted by maternal stress (5, 6), the composition of HM may also be affected by maternal stress.

Preterm HM has been shown to have higher levels of immune modulatory agents, such as secretory IgA (7), which is possibly protective against infection and inflammation. IgA amounts in HM are highly variable and vary between individuals, by health conditions, and over time. Nevertheless, these levels may be related to maternal stress, and the nature of these relationships remain poorly understood, especially in medically fragile premature infants and high-stress environments such as neonatal intensive care units (NICUs). Stress-responsive biological markers, such as cortisol, also vary in this way, though studies have demonstrated synchrony between maternal and breastfed infant salivary cortisol up to 12 months postpartum (8, 9).

Other research has linked maternal serum cortisol and secretory IgA with maternal mood and neonatal autonomic stability (10). These findings emphasize the potential to improve infant and maternal well-being by improving lactation support in the NICU and maximizing the benefits of HM composition. Some studies suggest that maternal stress reduction interventions may increase serum and HM secretory IgA levels (11), reduce subjective stress and salivary cortisol, and increase breastmilk production in NICU mothers (12).

Therefore, determining the impact of maternal stress on human milk (HM) and immunomodulatory agents may prove important for maximizing maternal and neonatal health. Specifically, we sought to determine: (1) whether reported measures of maternal stress were associated with immunologic markers in maternal circulation and HM and (2) whether HM composition was associated with infant health and neurodevelopment during NICU hospitalization.

## Materials and methods

### Study design

**Figure 1:**
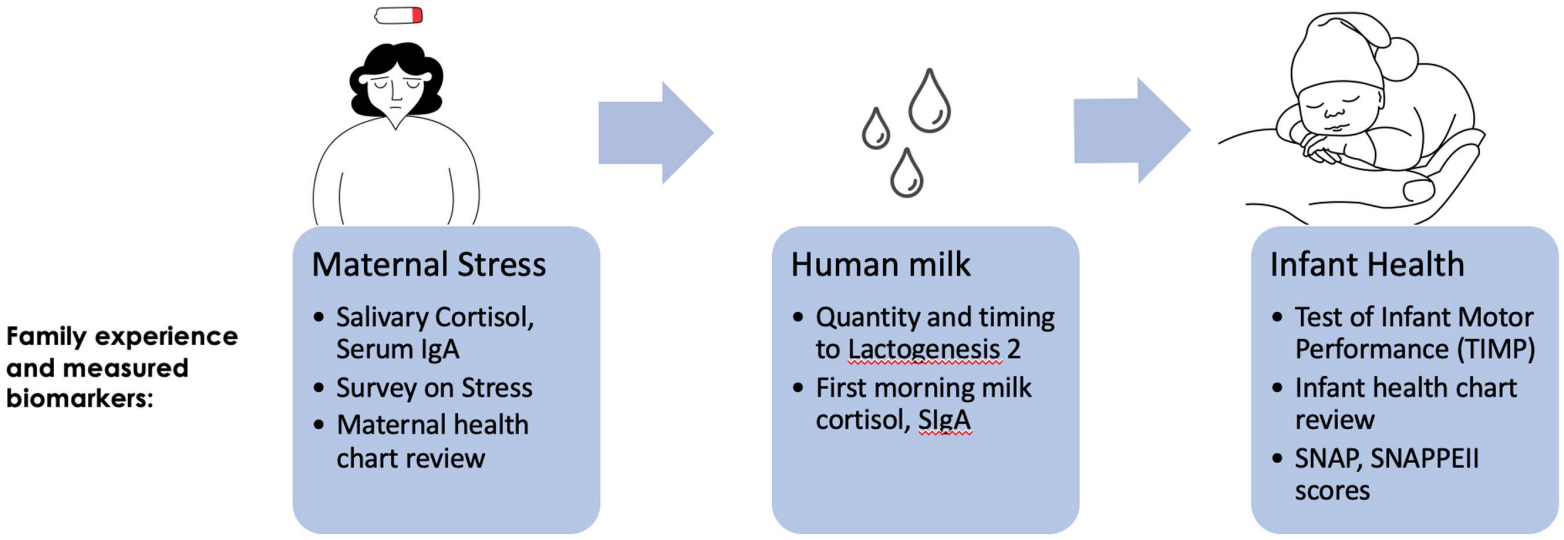
Flow diagram of the pilot study with measured biomarkers.

We conducted an observational cohort study at a Level 4 NICU that serves as a regional perinatal center. Our goal was to determine the trajectory of maternal stress (captured via surveys and biometric assays), its transmission to infants via HM (captured via composition assays), and relationships with infant outcomes (infant illness scores and neurodevelopmental testing; Figure 1).

We recruited dyads of mothers and moderately preterm infants (born at 28–32^6^/_7_ weeks gestation with an appropriate-for-gestational age birth weight). We approached mothers and lactating parents of infants, hospitalized in the NICU, who were less than 7-days old. The inclusion criteria included the intention to exclusively breastfeed, the mother’s routine pumping of breastmilk at least six times per day (indicating support for ongoing supply), and the absence of clinical indications for the supplementation of formula or donor milk at the time of enrollment. We excluded infants who were out of their mother’s legal custody, who had a medical contraindication to breastfeeding, and who received more than 50% of base feeds as donor human milk or infant formula at enrollment or discharge.

Maternal/infant dyads were seen for two study visits: at enrollment (week 1 postpartum) and 4 weeks post enrollment (week 5 postpartum).

### Sample collection

Maternal saliva samples were self-collected via spit collection at the time of the first-morning milk expression (5 a.m.–8 a.m.) on the day of the study visit. Mothers were given a salivary collection kit along with the instructions. Research assistants educated mothers on when and how to collect the sample. Saliva collection kits were brought back to the lab, where they were immediately spun at 10,000 g, and the supernatant was transferred to −80°C until analysis.

Human milk samples were self-collected by each mother before the day of the study visit. Mothers were provided a sterile collection kit and were instructed to perform a full-breast collection using their own electric breast pump (mothers expressed their breast completely until milk stopped flowing) at the time of their first daily expression (between 4 a.m. and 8 a.m.). Samples obtained outside this window were excluded from analysis. Upon expression, milk was swirled to homogenize, and a 5 mL of aliquot was removed and transferred to the lab. In the lab, 1 mL of the whole milk was spun at 10,000 g for 10 min at 4°C to separate fat from skim. A micropipette was used to pull skim from fat, using cold pipette tips to prevent fat from melting. The aliquot of skim milk, fat, and the remaining 4 mL of whole milk was then frozen at −80°C until analysis.

### Biological measures

#### Salivary cortisol

Cortisol was analyzed in duplicate as instructed using a commercially available chemiluminescent immunoassay (IBL Immuno-Biological Laboratories, Hamburg, Germany, catalog: RE62111).

#### HM cortisol

Milk cortisol was measured in whole milk following the method published by Hahn-Holbrook et al. (13) using an adapted salivary chemiluminescent immunoassay (IBL Immuno-Biological Laboratories, Hamburg, Germany, catalog: RE62111). In short, 200 μL of whole milk was thawed. A measure of 100 μL of sample was spiked with 2.5 ng of cortisol. Both spiked and un-spiked aliquots were then extracted by adding 500 μL of chilled dichloromethane to milk, vortexing and incubating on ice for 10 min. Samples were then centrifuged for 5 min at 1,500 g, and the top aqueous phase was removed and discarded. A measure of 100uL of the lower phase was transferred to a new tube and evaporated to dryness in a chemical fume hood. A measure of 50 μL of distilled water was used to resuspend the tube contents after sitting for 10 min at room temperature. Resuspended samples (both spiked and un-spiked) were analyzed in duplicate following the kit instructions, loading 20 μL of extraction per well. Relative luminescence units were measured within 10 min of assay completion. A four-parameter logistic curve was generated, and the results were corrected for extraction efficiency by adjusting according to the percent recovery of the spiked sample.

#### HM IgA

HM IgA was measured via custom immunoassay as previously described (14). In short, 96-well MaxiSorp plates were coated with 100 μL of anti-human IgA (Bethyl Lab A80–102A) at 1:100 dilution in 0.05 M carbonate–bicarbonate overnight at 4°C. The following day, the well contents were removed and the plate was blocked by adding 200 μL of 1% bovine serum albumin (BSA) in phosphate-buffered saline (PBS) for 30 min at room temperature. The blocking solution was removed, and the plate was washed for five cycles with a 300-μL wash of PBS-T solution containing 0.05% Tween-20 using a BioTek microplate washer. Then, the samples and controls were added to the plate for 2 h at room temperature. Skim milk was diluted at a concentration of 1:5,000 in 1% BSA in PBS before running, and the controls were prepared as a serial dilution of control serum (Bethyl Lab RS10–110) in 1% BSA in PBS. Following the incubation process, the plate was washed as described above, and then 100 μL of secondary antibody (Bethyl Lab A80–102P) was added at a concentration of 1:100,000 in 1% BSA in PBS for 1 h at room temperature. After this incubation, the plate was washed as described above, and then 100 μL of prepared TMB solution [BD Biosciences (Franklin Lakes, NJ) OptEIA TMB substrate reagent set (BD 555214)] was added to each well, followed by a 15-min incubation at room temperature in the dark. To stop the reaction, 50 μL of 0.18 sulfuric acid was added to each well, and the plate absorbance was read at 450 nm. A 5-parameter logistic curve was generated, and sample concentrations were calculated.

#### HM sodium and potassium

HM Na and K concentrations were measured using ion selective electrodes (Sodium: B-722; potassium: B-731; Horiba, Japan) as previously validated (15). In short, the electrodes were calibrated, and 300 μL of milk was added to the sensor for measurement. The samples were analyzed in duplicate. If duplicates were more than a 10% disparity, a triplicate measurement was performed. The sodium-to-potassium ratio (Na:K ratio) was calculated as a biomarker of lactogenesis 2 (16, 17).

### Interview/observational measures

Maternal demographics were collected at the time of enrollment. These included measures of maternal psychosocial and sociodemographic status (including income, education, family household composition, marital status, occupation, race, ethnicity, medical history, illness history, and medication use). Additionally, measures known to be associated with perinatal morbidity (e.g., hypertension, diabetes, and preterm labor) and breastfeeding difficulty (e.g., type of delivery, ICU stay, and infant separation) were collected by surveys and chart reviews (2, 3, 18–20). The demographic measures (listed above) were derived from the Centers for Disease Control and Prevention’s Pregnancy Risk Assessment Monitoring System and were adapted for use in Monroe County (21).

Maternal stress was measured at enrollment and at 4 weeks of age. Questionnaire measures of maternal stress included standard assessments of stress that had been previously used and validated in prior research (22, 23). These assessments comprised the Edinburgh Postnatal Depression Scale (EPDS), the Adverse Childhood Events Scale (ACES), the State–Trait Anxiety Inventory (STAI) (24–28), and the Patient-Reported Outcomes Measurement Information System (PROMIS) measures for sleep (29), and Cognitive Abilities Short Form 4a (30). We also included measures of social support, prenatal smoking, substance use, and health behaviors, such as physical activity (31). NICU-specific maternal stress indicators included the Parent Stress Scale: Neonatal Intensive Care Unit (PSS: NICU) (32) and the Postpartum Bonding Questionnaire (33, 34).

For breastfeeding measures, we collected information regarding time to first expression, time to first colostrum expressed, time to lactogenesis 2 (as per maternal report, defined by an increase in milk production > 15 mL/expression), exclusivity, weekly milk production, and time of first feed at the breast (35). Exclusivity was defined as the receipt of mother’s own only, with or without fortification.

Infant neurodevelopment was measured at 4 weeks of age or at discharge, whichever was first. The Test of Infant Motor Performance (TIMP) scale measures the cognitive and motor performance of premature infants (36, 37). This scale has been validated by the scale developers for use in research and was administered by a trained occupational therapist. Although initially validated for infants of 34 weeks gestation or more, this scale has been used for younger populations as well, including the changes in scores with maturation over time, which is the goal of our pilot study (22, 38, 39). Infant health was measured at enrollment and at 4 weeks of age. The Score for Neonatal Acute Physiology II (SNAP II) and the Score for Neonatal Acute Physiology with Perinatal Extension (SNAPPE II) are validated tools for determining the severity of illness and the mortality risk in newborns of all birthweights (40). The SNAP II evaluates the mortality risk from the severity of illness, including blood pressure, PO2/FiO2, lowest temperature, serum pH, multiple seizures, and urinary output. The SNAPPE II extends this evaluation to the perinatal period by including factors such as newborn weight, appearance, pulse, grimace, activity, and respiration (APGAR) score, and being small for gestational age to determine the mortality risk. Both scores are determined based on values obtained in the first 12 h of life.

### Statistical analysis

All statistical analyses were performed using SAS 9.4 (SAS Institute Inc., Cary, NC). Means, standard deviations, and ranges were used to summarize the continuous measures, while frequencies and percentages were used for categorical measures. Within patients, changes in HM and salivary cortisol as well as HM IgA over time were assessed using paired *t*-tests or Wilcoxon signed-rank tests, as appropriate. At each time point, Pearson’s correlation and Spearman correlation analyses were performed to evaluate associations between maternal stress and human milk cortisol and IgA, as appropriate. Due to the high skewness of cortisol in biological samples (35), HM and salivary cortisol were log-transformed in the analyses. Partial correlation coefficients were estimated after adjusting for gestational age (GA), corrected GA, and infant day of life (DOL). For binary stress variables, t-tests or Wilcoxon rank sum tests were used to evaluate the relationship between HM, salivary cortisol, and IgA. A *value of p* of <0.05 was considered statistically significant. No adjustment for multiple tests was made.

The sample size calculation was based on the ability to detect differences in maternal cortisol levels between stressed and non-stressed mothers (based on PSS) in the NICU. A sample of 25 achieves 80% power to detect an effect size of 1.2, assuming balanced group sizes at a two-sided significance level of 5%.

## Results

Cohort characteristics are presented in Table 1: 26 mothers (with a mean maternal age of 28.1 years, consisting of 69% white, 19% Black, and 8% Hispanic) and their infants (gestational age range: 28.5–33.5; SD = 1.8) were enrolled between May 2019 and March 2020. Time to lactogenesis 2 by parental report was collected. That lactogenesis 2 had occurred by the time of sample collection was verified by a Na:K ratio < 0.8 (35). Cortisol and IgA levels are described in Table 2. Mothers reported high levels of stress, postpartum mood disorders, anxiety, and sleep disorders, which were stable or worsened over time (Table 3).

**Table 1:** Subject characteristics.

Variable	*N* = 26
Maternal age^1^	28.1 years (range 18.5–38.7, SD 5.7)
**Race** ^**2**^	
White	69% (18)
Black	19% (5)
**Ethnicity**	
Hispanic	8% (3)
**Insurance type** ^**2**^	
Medicaid/Medicare	30.8% (8)
Private	65.4% (17)
Uninsured	3.8% (1)
Marital status^2^	53.85% married (14)
**Education** ^**2**^	
High school or less	30.7% (8)
Some college or more	69.23% (18)
Infant gestational age	Range 28.5–33.5 weeks; SD = 1.8
Infant SNAP II score^1^	6.5 (range 0–21, SD 5.66)
Infant SNAPPE II score^1^	8 (range 0–21, SD 6.15)
Time to first expression^1^	1.96 days (range − 0.31–8.15, SD 2.06)^3^
Time to L2 (by self-report)^1^	3.29 days (0–33 days, SD 6.49)
Time to first feed at the breast^1^	18.5 days (2–62, SD 16.7)

SD, standard deviation; L2, lactogenesis 2; SNAP II, Score for Neonatal Acute Physiology; SNAPPE II, Score for Neonatal Acute Physiology with Perinatal Extension-II. ^**1**^Reported as mean (range, standard deviation). ^2^Reported as % (*n*). ^3^Negative “time to first expression” represents antenatal hand expression.

**Table 2:** Human milk and salivary cortisol (raw and log-transformed) and human milk IgA^1^.

	1 week (*N* = 24)	5 weeks (*N* = 17)	Mean difference (*N* = 17)	*P*-value^2^
**Cortisol**				
Human milk cortisol	1.93 ng/mL (range 0.12–6.52, SD 1.97)	6.02 ng/mL (range 1.1–15.3, SD 3.9)	Mean 4.05 (range −0.6–14.74)	*p* < 0.001^*^
Human milk cortisol, log-transformed	0.10 (range −2.09–1.88, SD 1.19)	1.57 (range 0.09–2.73, SD 0.697)		0.0001^*^
Maternal salivary cortisol	2.25 ng/mL (range 0.04–9.24, SD 2.35)	2.43 ng/mL (range 0.48–6.86, SD 1.76)	Mean −0.13 (range −5.37–3.76)	0.8
Maternal salivary cortisol, log-transformed	0.117 (range −3.22–2.22, SD 1.52)	0.56 (range −0.798–1.925)		0.17
**IgA**				
Breastmilk IgA	1.13 mg/mL (range 0.42–1.91, SD 0.47)	0.89 mg/mL (range 0.25–1.86, SD 0.45)	−0.145 mg/mL (range −0.86–0.89, SD 0.53)	<0.0001^*^

^*^Statistically significant. ^1^HM and salivary cortisol was measured using chemiluminescent immunoassay, and IgA was measured using customized immunoassay in skim milk. ^2^For comparison over time.

**Table 3:** Maternal stress measures over time (week 1 to week 5).

Stress measure	Week 1, *N* = 25	Week 5 *N* = 21	*P*-value^1^
PSS:NICU^2^ “How stressful has the experience of having your baby hospitalized been for you?”	4.47 (very stressful, range 2–6, SD 1.2)	4.87 (3–6, SD 1.14)	0.36
PSS:NICU mean all questions combined	3.37 (1.7–5.9, SD 1.15)	3.31 (1.8–5.6, SD 1.13)	0.81
EPDS^3^	12.3 (3–24, SD 5.34)	12.18 (3–27, SD 6.82)	0.23
EPDS^3^ score > 13, depression	46% of participants (*n* = 12 of 26)	36% of participants (*n* = 8 of 22)	0.69
Sleep^4^, moderate to severe sleep difficulty	68% (17)	52.4% (11)	0.18
Sleep^4^, T-score	62.3 (36.6–78.5, SD 12.98)	60.5 (36.6–80.3, SD 9.9)	0.07
Maternal anxiety (STAI^5^)	36 (20–64, SD 10.9)	39 (23–73, SD 16.01)	0.21
Maternal high anxiety (STAI^5^ score > 53)	8% of participants (*n* = 2)	23% of participants (*n* = 5)	0.0006^*^

Results reported as mean (range, standard deviation). SD = standard deviation. ^*^Statistically significant. ^1^For comparison over time. ^2^Parental Stressor Scale: Neonatal Intensive Care Unit. ^3^Edinburgh Postnatal Depression Scale. ^4^Adult PROMIS—Sleep Disturbance—Short Form, Patient-Reported Outcomes Measurement Information System. Raw scores are converted to T-scores based on patient response rate. T-scores are used for interpretation. A score of 55 or below indicates no to mild sleep disturbance, a score over 60 indicates moderate sleep disturbance, while a score of 70 and over indicates severe sleep disturbance. ^5^State–Trait Anxiety Inventory.

### Timing of lactogenesis 2

No demographic variables, markers of maternal stress, or HM analytes correlated with time to lactogenesis 2 (as reported by the patient or by the Na:K ratio).

### Cortisol

Neither maternal age, parity, infant gestational age nor time to lactogenesis 2 was associated with HM cortisol concentrations.

**Figure 2:**
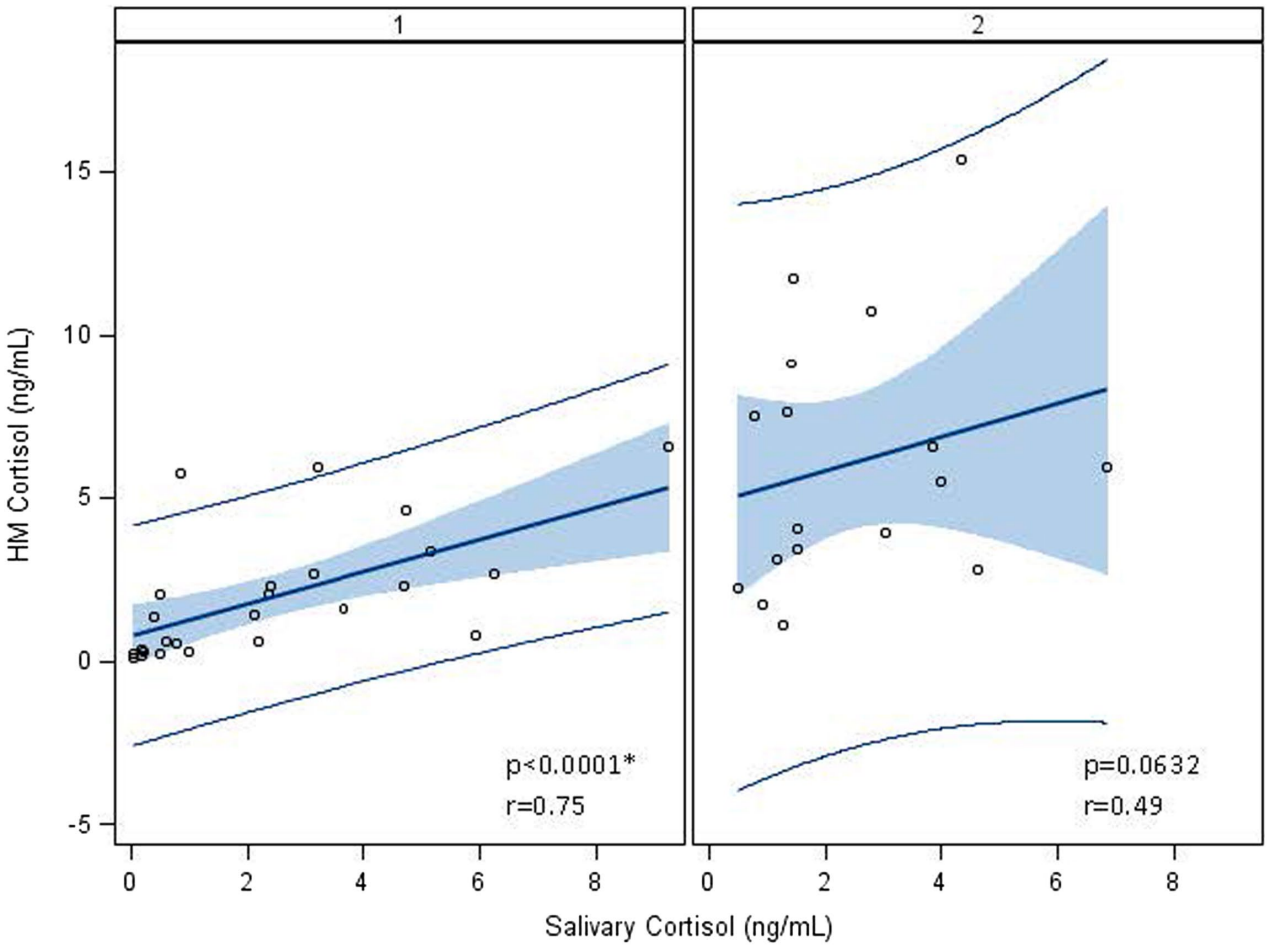
Association between log-transformed human milk and salivary cortisol over time, from visit 1 (week 1) to visit 2 (week 5). At visit 1 (week 1 postpartum), human milk (HM) cortisol was associated with maternal salivary cortisol. At visit 2 (week 5 postpartum), this association no longer held, and cortisol measures were log-transformed to account for the skewness of cortisol in biological samples. ^*^Statistically significant.

Maternal salivary cortisol remained stable over time (Table 2; 2.25 ± 2.35 ng/mL–2.43 ± 1.77 ng/mL, *p* = 0.17). HM cortisol increased from 1.93 ± 1.97 ng/mL at week 1 to 6.03 ± 3.93 ng/mL at weeks 5 (*p* < 0.0001). Human milk and salivary cortisol were closely associated in week 1 but not in week 5 (Table 4; Figure 2). Measures of maternal stress were associated with HM cortisol at week 1 but not at week 5 (Table 4). No associations were found between EPDS, sleep, ACES, and PSS:NICU other categories and either HM or salivary cortisol at both time points. PROMIS clarity of thought at week 1 was positively associated with maternal salivary cortisol. The change in anxiety (STAI) between weeks 1 and 5 was positively associated with the increase in HM cortisol, but this association did not hold for salivary cortisol (Table 4).

**Table 4:** Associations between maternal stress and human milk cortisol and IgA over time (week 1 to week 5).

Associations with HM cortisol (log-transformed)		
Independent variable	Week 1	Week 5
Maternal Salivary Cortisol (log-transformed)	r = 0.75	r = 0.49
	*p* < 0.001^*^	*p* = 0.06
PSS:NICU^1^ sub-score, stress associated with parental role	r = − 0.44	r = 0.22
	*p* = 0.047^*^	*p* = 0.44
PROMIS^3^: clarity of thought	r = 0.54	r = −0.06
	*p* = 0.01^*^	*p* = 0.83
Maternal anxiety (STAI^2^)	r = −0.40	r = 0.18
	*p* = 0.007^*^	*p* = 0.52
Change in HM Cortisol vs. Change in Maternal Anxiety (STAI^2^)	r = 0.58	
	*p* = 0.03^*^	
**Associations with HM IgA**		
**Independent variable**	**Week 1**	**Week 5**
PSS: NICU^1^ score, overall stress with hospitalization	r = 0.35	r = 0.79
	*p* = 0.18	*p* ≤ 0.001^*^
Change in PSS:NICU^1^ score, overall stress with hospitalization vs. Change in HM IgA	r = 0.70	
	*p* = 0.02	

HM, human milk. ^*^Statistically significant. ^**^Higher score = worse outcomes associated with higher mortality rates. ^1^Parental Stressor Scale: Neonatal Intensive Care Unit. ^2^State–Trait Anxiety Inventory. ^3^Patient-Reported Outcomes Measurement Information System. ^4^Score for Neonatal Acute Physiology.

No associations were found between maternal salivary or HM cortisol and infant illness scores at any time point (SNAP II and SNAPPE II; data not shown).

### IgA

**Figure 3:**
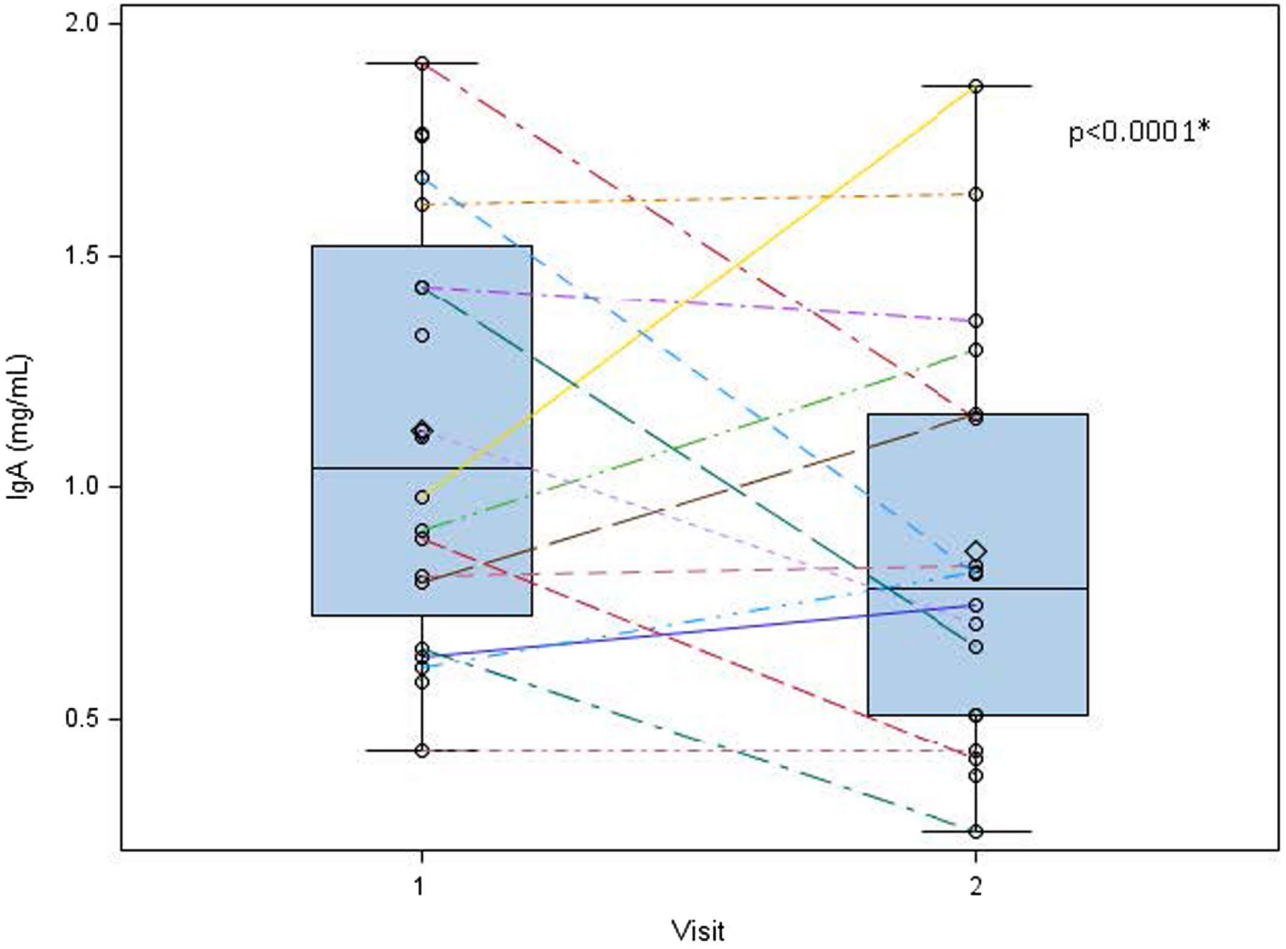
Human milk IgA decreases overtime from 1 to 5 weeks postpartum. Human milk IgA was measured at visit 1 (week 1 postpartum) and visit 2 (week 5 postpartum). While significant variation exists in human milk IgA between subjects, we found a general decreasing trend in these measures from week 1 to week 5 postpartum. 0 = mean value, the center line denotes the median value, the box contains the 25th to 75th percentile of the dataset, and the black whiskers mark the minimum and maximum values. ^*^Statistically significant.

**Figure 4:**
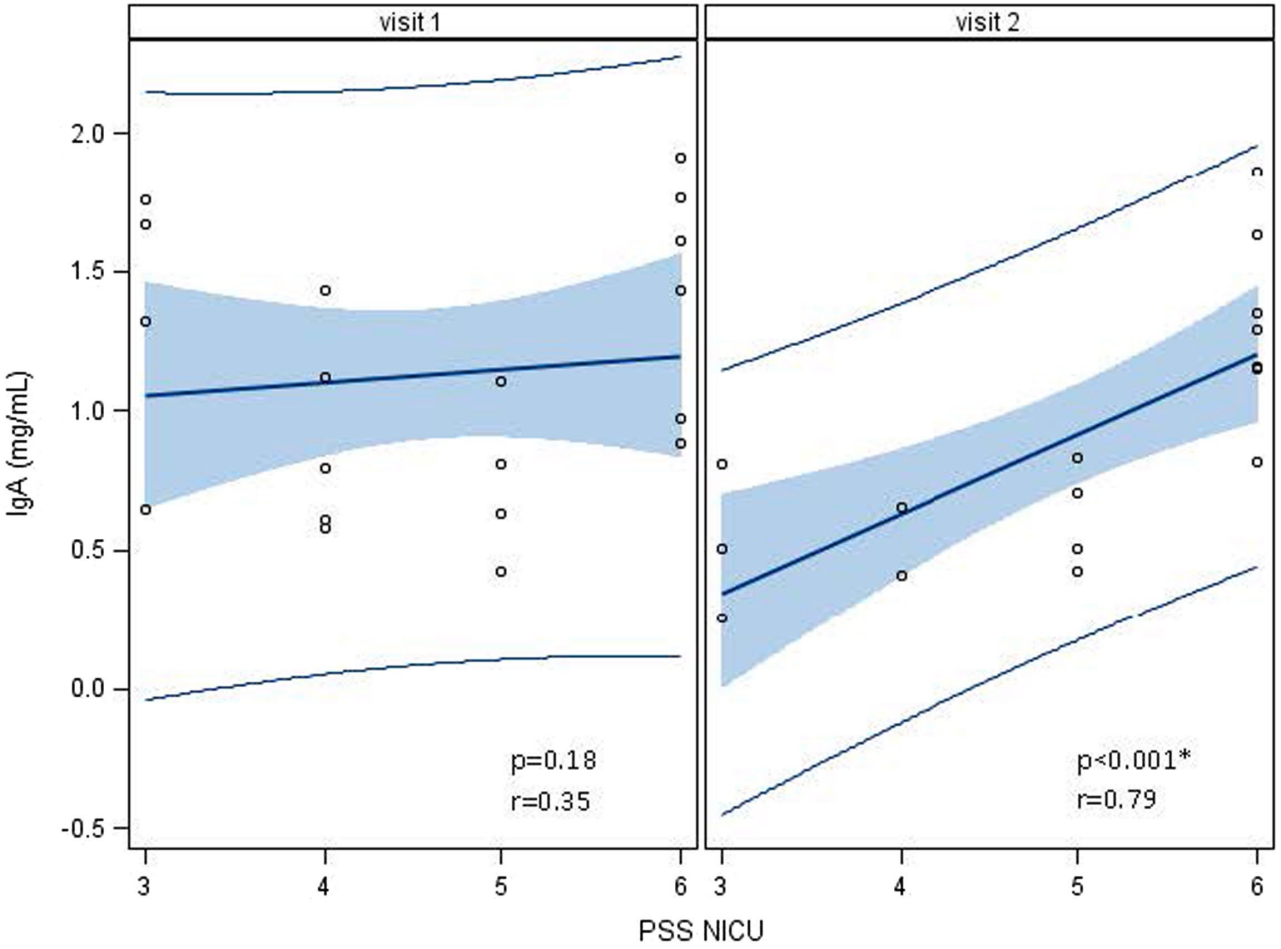
Associations between human milk IgA and the Parent Stress Scale: Neonatal Intensive Care Unit (PSS: NICU) over time, visit 1 (week 1) to visit 2 (week 5). At visit 1 (week 1 postpartum), human milk (HM) IgA was not associated with a high maternal stress score on the PSS:NICU. At visit 2 (week 5 postpartum), this association was significant. ^*^Statistically significant.

Human milk IgA decreased over time from 1.13 ± 0.47 mg/mL at 1 week to 0.89 ± 0.45 mg/mL at 5 weeks (*p* < 0.0001; Table 2; Figure 3). Neither maternal age, parity, infant gestational age nor time to lactogenesis 2 was associated with HM IgA concentrations. High stress on the PSS:NICU scale at week 5 was associated with HM IgA (Table 4; Figure 4). The change in stress (via PSS:NICU score, overall stress with hospitalization) was positively associated with the change in HM IgA (Table 4; Figure 4).

HM IgA at week 1was negatively associated with infant SNAP II score at week 1 (r = −0.75, *p* = 0.05); no associations were found between HM IgA and SNAPPE II. No other associations with maternal stress measures were found.

### Neurodevelopment

No associations were found between maternal stress, salivary cortisol, HM cortisol, or HM IgA and neurodevelopment at discharge (TIMP; data not shown). These relationships did not differ by infant sex.

## Discussion

In our study of mothers of NICU infants over the first 5 weeks of hospitalization, maternal salivary cortisol remained stable, while HM cortisol increased over time. However, with the exception of the PROMIS clarity of thought scale, maternal anxiety and stress were negatively associated with HM *but not* with salivary cortisol. These associations were found during the early postpartum period (week 1) but were not evident 1 month later (week 5). Conversely, HM IgA decreased over time. At week 5, maternal stress on the PSS:NICU scale was positively associated with HM IgA, suggesting that mothers with higher stress had higher levels of IgA in their milk. HM IgA was in turn negatively associated with the infant illness score (SNAP II) at week 1, indicating that higher IgA was associated with lower SNAP II scores (a healthier infant).

### HM cortisol trends and relationship between salivary and milk cortisol

This positive correlation between maternal salivary and HM cortisol has been reported in other studies involving term and preterm infants (41–43). However, Romijn et al. detected this association in term infants at 1 month postpartum, unlike our analysis, in which the association dissipated over time. Other studies have found that HM cortisol concentrations are highest in the colostral phase and decrease in mature milk after term birth (44, 45). This finding contrasts with the studies of preterm milk over time, which show higher cortisol levels compared to term milk (46, 47), with levels increasing over time (47). These conflicting reports may represent differences in cortisol secretion in term vs. preterm milk and have differing impacts on the recipient infant. The concentrations of HM cortisol detected in our study (collected from a full-breast expression) were comparable to those reported in other preterm milk studies and lower than those reported in studies that collected hindmilk samples (42, 47, 48).

### HM IgA levels and trends

IgA levels in our study were similar to those observed in other studies, which have been reported at 1.6–2 mg/mL in transitional and mature milk (49). Though IgA levels are typically higher in preterm milk, this difference is more pronounced in colostrum, which we did not collect (7). Human milk IgA decreased from weeks 1 to 5; however, associations between maternal stress and IgA were mostly stable over time. This decrease in HM IgA over time has been reported in other studies (50, 51). There is significant variation in HM IgA concentration among individuals. The milk IgA content further varies by age, parity, mode of delivery, BMI, gestational age, and over time (52–54). IgA is greater in HM from the mothers of infants <1,000 g and may be higher in male infants (55). However, we detected no difference in HM composition based on infant sex in this study. Some research has found that stress and maternal mood influence HM secretory IgA (56). There are significant data to suggest that HM IgA provides passive protection to infants against specific infectious diseases (57–59), and also shapes the developing microbiome (49, 60, 61).

### HM cortisol and IgA and stress

We detected a negative association between maternal stress (PSS:NICU) and anxiety and HM cortisol in the early postpartum period. However, as anxiety increased over the course of hospitalization, HM cortisol levels also rose. Other studies have reported associations between maternal stress and depression scores and glucocorticoids in HM and serum (46, 47, 62, 63), while others fail to detect a relationship (64). IgA in HM is more consistently associated with subjective stress measures, as observed in our study, with higher levels of stress on the PSS:NICU scale associated with higher IgA levels in milk (56, 63, 65, 66). Moreover, when analyzed individually, changes in overall stress with hospitalization (PSS:NICU) were positively associated with changes in HM IgA. In addition to being linked to subjective stress measures, concentrations of HM cortisol and IgA are also modifiable. Studies on relaxation, meditation, and laughter have been shown to increase HM IgA and lower HM cortisol (11, 67, 68). These studies, although small and heterogeneous, suggest that alternative factors contribute to the variation in HM cortisol and IgA. This may, in turn, help to explain the variability in these associations between different studies.

### Relationships between HM cortisol, IgA, and infant outcomes

Both maternal serum and human milk cortisol appear to have effects on recipient infants. High maternal serum and hair cortisol levels have been associated with delayed lactogenesis 2, lower breastfeeding rates, and milk composition (69–72). Human milk cortisol is associated with infant biometrics (adiposity and head circumference), temperament, and fear reactivity (73–78). We therefore sought to determine if HM cortisol or HM IgA in this highly vulnerable preterm infant population was associated with neurodevelopment. However, our findings indicated no significant association. The findings reported in our study are similar to a study of healthy term infants in whom HM cortisol had no impact on temperament or neurodevelopment (76). We also did not detect any associations between maternal stress and the infant TIMP score.

In this population of preterm infants, we report that HM IgA is negatively associated with the infant illness score (SNAP II), suggesting that mothers with higher concentrations of HM IgA had infants with healthier illness scores. Alterations in HM IgA can have important ramifications for infant immune outcomes. This study is observational and thus cannot establish causality. Furthermore, though the SNAP II score includes variables affected by infant infection (blood pressure, PO2/FiO2, lowest temperature, serum pH, multiple seizures, and urinary output), it is not specific to infectious illness. No association was found between HM IgA and the SNAPPE II, which includes perinatal mortality indicators (such as newborn weight, APGAR score, and being small for gestational age) that are not illness-specific. We therefore postulate that these data support the premise that higher HM IgA may be a protective factor against infant infectious disease. This finding warrants further validation and mechanistic investigation, especially in premature infants.

## Strengths and limitations

The strengths of this study included the assessment of an understudied population (premature, hospitalized infants), the characterization of maternal stress using multiple assessments, the inclusion of multiple specimen types, including saliva and HM, gathered contemporaneously, and the use of validated assessments to evaluate both infant neurodevelopment and illness.

The limitations of the study include the small sample size (which was limited due to the COVID-19 pandemic) and the lack of infant biomarker analysis. This limited sample size reduced our power to detect relationships with a small effect size. Thus, our inability to detect any relationship between maternal stress, HM cortisol, or HM IgA and infant neurodevelopment could represent a true verification of the null hypothesis or reflect our limited power. It could also affect our findings regarding the direction of the association between HM cortisol and reported maternal stress.

The small sample size also limited our ability to control for demographic and medical stressors, such as income, race, marital status, maternal illness, and antenatal steroid use, which may impact breastfeeding outcomes, cortisol levels, or infant neurodevelopment.

We used salivary cortisol as an objective measure of stress instead of serum cortisol, due to its lower circadian variability and the ability to collect the sample at the same time as milk expression on first-morning awakening. However, salivary cortisol levels in the peripartum period may be affected by an impaired adrenal capacity to mount a cortisol response and variable cortisol metabolism, which were not documented (79).

## Conclusion

Reported stress in NICU mothers was more tightly correlated with HM than salivary cortisol at 1 week postpartum. Maternal stress was associated with higher HM IgA levels, which, in turn, were associated with lower measures of infant illness.

Therefore, implementing stress reduction techniques for NICU mothers may have significant implications for the health of sick neonates.

## Data availability statement

The raw data supporting the conclusions of this article will be made available by the authors, without undue reservation.

## Ethics statement

The studies involving humans were approved by the Institutional Review Board of the University of Rochester. The studies were conducted in accordance with the local legislation and institutional requirements. Written informed consent for participation in this study was provided by the participants themselves, and participants’ legal guardians/next of kin.

## Author contributions

CR-C: Conceptualization, Formal analysis, Funding acquisition, Investigation, Methodology, Project administration, Resources, Supervision, Writing – original draft, Writing – review & editing. SG: Project administration, Writing – review & editing. HW: Data curation, Formal analysis, Writing – review & editing. SS: Investigation, Writing – review & editing. RM: Methodology, Writing – review & editing. TO’C: Conceptualization, Methodology, Writing – review & editing. KJ: Conceptualization, Methodology, Writing – review & editing. CD’A: Conceptualization, Funding acquisition, Methodology, Writing – review & editing. BY: Formal analysis, Investigation, Supervision, Writing – original draft, Writing – review & editing.
